# ChAdOx1-vectored Lassa fever vaccine elicits a robust cellular and humoral immune response and protects guinea pigs against lethal Lassa virus challenge

**DOI:** 10.1038/s41541-021-00291-x

**Published:** 2021-03-02

**Authors:** Robert J. Fischer, Jyothi N. Purushotham, Neeltje van Doremalen, Sarah Sebastian, Kimberly Meade-White, Kathleen Cordova, Michael Letko, M. Jeremiah Matson, Friederike Feldmann, Elaine Haddock, Rachel LaCasse, Greg Saturday, Teresa Lambe, Sarah C. Gilbert, Vincent J. Munster

**Affiliations:** 1 Laboratory of Virology, Division of Intramural Research, National Institute of Allergy and Infectious Diseases, National Institutes of Health, Rocky Mountain Laboratories, Hamilton, MT USA; 2 The Jenner Institute, Nuffield Department of Medicine, University of Oxford, Oxford, UK; 3 Rocky Mountain Veterinary Branch, Division of Intramural Research, National Institute of Allergy and Infectious Diseases, National Institutes of Health, Hamilton, MT USA; 4 Paul G. Allen School of Global Animal Health, Washington State University, Pullman, WA USA; 5 Marshall University Joan C. Edwards School of Medicine, Huntington, WV USA; 6 Present Address: Vaccitech Limited, Oxford, UK

**Keywords:** Virology, Microbiology, Vaccines, Immunology, Diseases

## Abstract

Lassa virus (LASV) infects hundreds of thousands of individuals each year, highlighting the need for the accelerated development of preventive, diagnostic, and therapeutic interventions. To date, no vaccine has been licensed for LASV. ChAdOx1-Lassa-GPC is a chimpanzee adenovirus-vectored vaccine encoding the Josiah strain LASV glycoprotein precursor (GPC) gene. In the following study, we show that ChAdOx1-Lassa-GPC is immunogenic, inducing robust T-cell and antibody responses in mice. Furthermore, a single dose of ChAdOx1-Lassa-GPC fully protects Hartley guinea pigs against morbidity and mortality following lethal challenge with a guinea pig-adapted LASV (strain Josiah). By contrast, control vaccinated animals reached euthanasia criteria 10–12 days after infection. Limited amounts of LASV RNA were detected in the tissues of vaccinated animals. Viable LASV was detected in only one animal receiving a single dose of the vaccine. A prime-boost regimen of ChAdOx1-Lassa-GPC in guinea pigs significantly increased antigen-specific antibody titers and cleared viable LASV from the tissues. These data support further development of ChAdOx1-Lassa-GPC and testing in non-human primate models of infection.

## Introduction

Lassa virus (LASV), an Old World arenavirus, was first identified in Nigeria in 1969^1^. Since then, LASV infections have been documented in countries of the Mano River Union (Sierra Leone, Guinea, Libera, Côte d’Ivoire), Mali, and Nigeria^2–4^. Isolated cases have also been reported in Benin and Togo^5^. Human infections occur after exposure to the urine or feces of infectious rodents, namely *Mastomys natalensis* (natal multimammate rat), the primary reservoir of LASV^6,7^. Human-to-human transmission of LASV has been documented, and is most commonly nosocomial^8,9^.

The burden of LASV infection remains poorly characterized. Although an annual incidence of 100,000–300,000 cases is often cited, this figure has been extrapolated from a single longitudinal study published in 1987 and has not been updated to reflect increases in population and diagnostic capability^10,11^. LASV infection is often asymptomatic in endemic areas. However, in some individuals, it causes an acute febrile illness—Lassa fever—ranging in severity from mild to an often-fatal multisystem syndrome. Case fatality rates during outbreaks, among hospitalized patients, and during the third trimester of pregnancy are reported to be 50%, 70%, and 90%, respectively^12–16^. Survivors of Lassa fever may contend with chronic sequelae like sensorineural hearing loss, which is observed following nearly 25% of symptomatic infections^17,18^.

At least seven distinct phylogenetic lineages of LASV exist. Lineages I–III circulate in Nigeria, while Lineage IV strains are distributed across Sierra Leone, Guinea, and Liberia^2,19–21^. Strains isolated from Côte d’Ivoire and Mali comprise lineage V^22^. The new Kako strain isolated from *Hylomyscus pamfi* rodents represents lineage VI and a virus isolated during an outbreak in Togo constitutes lineage VII^5,23^.

Currently, there is no licensed vaccine for Lassa fever, although numerous candidates are in the development pipeline. These include DNA, RNA, live attenuated, and multiple different viral-vectored vaccine approaches^24–32^. The protective efficacy of these vaccine candidates has been demonstrated in rodent and non-human primate models of LASV infection. To date, two candidates—plasmid DNA delivered via dermal electroporation and a measles virus (MV) vectored vaccine—have advanced to evaluation in phase 1 clinical trials^24,32^. The World Health Organization (WHO) and the Coalition for Epidemic Preparedness Innovations (CEPI) have listed LASV as a priority pathogen for vaccine development, which will support the progression of other candidates to clinical assessment in the near future.

ChAdOx1 is a chimpanzee adenovirus vector platform that has been employed in the development of vaccine candidates for a diverse series of pathogens. A single dose of ChAdOx1-vectored vaccine fully protects against Rift Valley fever virus, Middle East respiratory syndrome coronavirus, Zika virus, and *Mycobacterium tuberculosis* in animal models of infection^33–37^. In this study, we report on the preclinical immunogenicity and efficacy of a ChAdOx1-vectored Lassa fever vaccine encoding the full-length Josiah strain LASV glycoprotein precursor (GPC) sequence, ChAdOx1-Lassa-GPC. Immunogenicity was evaluated in mice and particular focus was given to the characterization of the T-cell response, including cross-reactivity to distinct strains. Protective efficacy after a single dose or prime-boost vaccine regimen was determined in a guinea pig model of lethal Lassa fever^38^. ChAdOx1-Lassa-GPC has been selected by CEPI as a promising candidate warranting accelerated development.

## Results

### ChAdOx1-Lassa-GPC is immunogenic and induces a polyfunctional T-cell response in mice

**Fig. 1:**
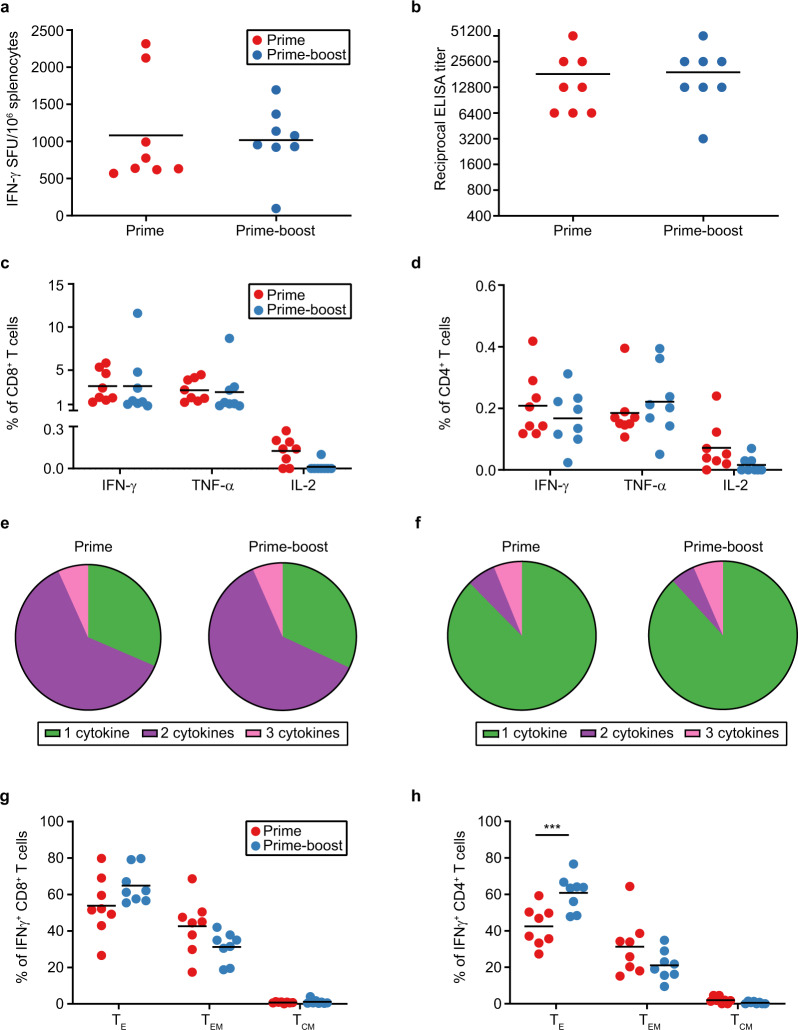
ChAdOx1-Lassa-GPC vaccination elicits a potent cellular and humoral immune response in mice. CD-1 mice received prime (*n* = 8) or prime-boost (*n* = 8) vaccination with ChAdOx1-Lassa-GPC. Spleen and serum samples were collected three weeks after the final immunization. **a** IFN-γ ELISpot of murine splenocytes measured in spot forming units (SFU) per 1.0 × 10^6^ splenocytes. The magnitudes of the prime and prime-boost responses were determined to be statistically similar by an unpaired *t*-test. **b** LASV GP-specific IgG antibody titers in twofold serial-diluted sera were measured by ELISA and were statistically similar according to Mann–Whitney test. The percentage of CD8^+^ (**c**) and CD4^+^ (**d**) T cells expressing IFN-γ, TNF-α, or IL-2 after GPC peptide pool stimulation was determined by intracellular cytokine staining of splenocytes. Graphed values reflect adjustment to exclude non-specific cytokine expression as measured from a corresponding set of unstimulated samples. All statistical comparisons between the prime and prime-boost vaccinates were not significant by two-way ANOVA with Sidak’s multiple comparisons test. The proportion of cytokine-secreting (IFN-γ^+^, TNF-α^+^, or IL-2^+^) CD8^+^ (**e**) and CD4^+^ (**f**) T cells expressing only one or multiple cytokines. Percentages of LASV-specific (IFN-γ^+^) CD8^+^ (**g**) and CD4^+^ (**h**) T cells exhibiting T_E_, T_EM_, and T_CM_ phenotypes as determined by immunostaining for CD44, CD62L, and CD127 surface markers. Phenotypic differences in the T-cell responses of the prime and prime-boost groups were not statistically significant by two-way ANOVA with Sidak’s multiple comparisons test, except for the increase in the CD4^+^ T_E_ subset post boost (***p = 0.0009).

Evaluation of the immunogenicity of ChAdOx1-Lassa-GPC was carried out in CD-1 mice. ChAdOx1-Lassa-GPC was administered intramuscularly as either a single dose or prime-boost vaccination regimen (*n* = 8 per group), with a 28-day interval between doses (Supplemental Fig. 1). After 21 days, LASV-specific T-cell and antibody responses were measured by IFN-γ ELISpot (Fig. 1a) and IgG ELISA (Fig. 1b), respectively. The magnitude of the IFN-γ T-cell response was determined by restimulating splenocytes with a pool of overlapping peptides spanning the full-length Josiah strain LASV GPC sequence. The mean response after a single dose of ChAdOx1-Lassa-GPC was not significantly amplified by delivery of a second dose (mean ± SEM: prime = 1083 ± 253, prime-boost = 1023 ± 162 SFU/10^6^ splenocytes). Although mean serum IgG antibody titers to Josiah strain LASV glycoprotein (GP) were elevated in prime-boost vaccinates compared to prime vaccinates, this difference was not statistically significant (mean ± SEM ELISA titer: prime = 18400 ± 5468, prime-boost = 21200 ± 5165). These assays illustrate induction of robust cellular and humoral immune responses after a single vaccination with ChAdOx1-Lassa-GPC.

There is some evidence suggesting that the resolution of LASV infection may principally be mediated by the cellular immune response; therefore, we further characterized LASV-specific CD8^+^ and CD4^+^ T cells by flow cytometric analysis^12,39–41^. (The gating strategy employed is depicted in Supplemental Fig. 2.) T-cell activation was assessed by measuring the expression of proinflammatory cytokines—IFN-γ, TNF-α, and IL-2. Intracellular cytokine staining was carried out on splenocytes restimulated with a LASV GPC peptide pool. In the CD8^+^ T cells profiled, IFN-γ was expressed with the highest mean frequency (mean percentage of CD8^+^ T cells: prime = 3.13%, prime-boost = 3.13%), followed by TNF-α (mean percentage of CD8^+^ T cells: prime = 2.66%, prime-boost = 2.44%) (Fig. 1c). At the timepoint of detection, the proinflammatory cytokine response in CD4^+^ T cells was lower in comparison to CD8^+^ T cells (mean percentage of CD4^+^ T cells secreting IFN-γ: prime = 0.21%, prime-boost = 0.17%; mean percentage of CD4^+^ T cells secreting TNF-α: prime = 0.19%, prime-boost = 0.22%) (Fig. 1d). Expression of IL-2 was observed in a minimal subset of CD8^+^ and CD4^+^ T cells (mean percentage of CD8^+^ T cells: prime = 0.13%, prime-boost = 0.01%; mean percentage of CD4^+^ T cells: prime = 0.07%, prime-boost = 0.02%). Comparisons of mean cytokine expression after delivery of a single dose or prime-boost regimen of ChAdOx1-Lassa-GPC were not statistically significant.

Over 60% of vaccine-induced IFN-γ^+^, TNF-α^+^, or IL-2^+^ CD8^+^ T cells expressed more than one cytokine, indicating the development of a polyfunctional response (proportion of cytokine-secreting CD8^+^ T cells expressing 1, 2, or 3 cytokines: prime = 31.47%, 61.80%, 6.73%; prime-boost = 31.94%, 61.45%, 6.60%) (Fig. 1e). The majority of polyfunctional T cells co-expressed IFN-γ and TNF-α (data not shown). By contrast, the small subset of cytokine-producing CD4^+^ T cells was largely monofunctional, expressing either IFN-γ or TNF-α (proportion of cytokine-secreting CD4^+^ T cells expressing 1, 2, or 3 cytokines: prime = 87.60%, 6.26%, 6.15%; prime-boost = 88.17%, 5.35%, 6.48%) (Fig. 1f). Polyfunctionality of CD8^+^ and CD4^+^ T cells was not further enhanced by boosting.

Surface phenotyping of CD8^+^ and CD4^+^ T cells revealed predominantly effector and effector memory subsets. IFN-γ expression was used to distinguish LASV-specific T cells. Antigen-experienced effector and memory cells were further distinguished by surface expression of CD44, a marker of T-cell activation. Effector (T_E_), effector memory (T_EM_), and central memory (T_CM_) populations were classified based on surface expression of CD62L (L-selectin, lymphoid trafficking marker) and CD127 (IL-7 receptor, homeostatic survival marker) as follows: CD44^+^ CD62L^−^ CD127^−^ (T_E_), CD44^+^ CD62L^−^ CD127^+^ (T_EM_), and CD44^+^ CD62L^+^ CD127^+^ (T_CM_). T_E_ and, to a lesser extent, T_EM_ phenotypes comprised the IFN-γ^+^ CD8^+^ T-cell response (mean percentage of IFN-γ^+^ CD8^+^ T cells with T_E_, T_EM_, or T_CM_ phenotype: prime = 53.84%, 42.61%, 0.73%; prime-boost = 64.84%, 31.24%, 1.09%) (Fig. 1g). Similar results were observed for IFN-γ^+^ CD4^+^ T cells (mean percentage of IFN-γ^+^ CD4^+^ T cells with T_E_, T_EM_, or T_CM_ phenotype: prime = 42.44%, 31.29%, 2.01%; prime-boost = 60.86%, 21.12%, 0.51%) (Fig. 1h). The T_CM_ phenotype was largely absent from the CD8^+^ and CD4^+^ T cells profiled. This finding is consistent with previous reports indicating that the kinetics of the T_CM_ response following adenovirus vaccination are slower than that for some other viral vectors and are only detectable a few months after immunization^42,43^. Although the effect was only statistically significant for CD4^+^ T-cell subsets, administration of a second dose of ChAdOx1-Lassa-GPC appeared to favor greater proliferation of both CD8^+^ and CD4^+^ T_E_ cells, and a proportional reduction in T_EM_ cells (*p* = 0.0009, two-way ANOVA with Sidak’s multiple comparisons test). Together, these data indicate that vaccination with ChAdOx1-Lassa-GPC induces a fully differentiated, polyfunctional T-cell response. Three weeks after immunization, cellular immunity is dominated by effector and effector memory mechanisms. Furthermore, significant differences in the magnitude or quality of the T-cell response are largely not observed between single dose and prime-boost vaccination regimens.

### Vaccine-induced T cells and IgG antibodies cross-react with GP from diverse LASV strains

**Fig. 2:**
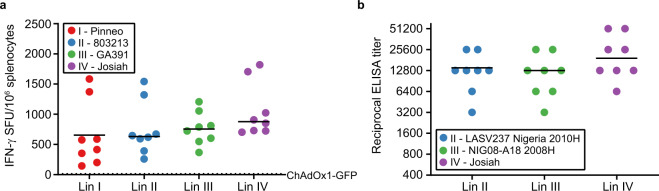
Vaccine-specific IFN-γ^+^ T cells and IgG antibodies exhibit cross-reactivity to GP from heterologous LASV strains. **a** CD-1 mice were vaccinated with ChAdOx1-Lassa-GPC (*n* = 8) or ChAdOx1-GFP (*n* = 2) and euthanized after 14 days for the collection of splenocytes. IFN-γ-secreting T cells were quantified by ELISpot assay after stimulation with four different peptide pools corresponding to the full-length GPC sequence from a representative strain from LASV lineages I–IV. Responses are reported as spot forming units (SFU) per 1.0 × 10^6^ splenocytes. The dotted line indicates the mean response in ChAdOx1-GFP vaccinates. **b** CD-1 mice were vaccinated with ChAdOx1-Lassa-GPC (*n* = 8) or ChAdOx1-GFP (*n* = 2) and euthanized after 28 days for sera collection. IgG antibody titers against GP from representative strains from LASV lineages II–IV were measured in twofold serial-diluted sera by ELISA. Josiah strain GP (lineage IV) is homologous to the vaccine antigen. The magnitudes of IFN-γ^+^ T-cell responses and titers of IgG antibodies displaying reactivity to each heterologous strain were determined to be statistically comparable to Josiah strain-specific responses by one-way ANOVA with Dunnett’s multiple comparisons test.

The ability to confer protection against infection by genetically diverse LASV strains will be a critical feature of an effective Lassa fever vaccine. Circulating strains may vary up to 32% and 25% in the genomic L and S segments, respectively^44^. ChAdOx1-Lassa-GPC encodes antigen derived from the canonical lineage IV Josiah strain virus. Prior to progressing to heterologous LASV challenge experiments in guinea pigs, we verified that vaccine-induced T-cell and antibody responses exhibited cross-reactivity to GP from multiple LASV lineages. CD-1 mice were immunized with ChAdOx1-Lassa-GPC (*n* = 8) or ChAdOx1-GFP control vaccine (*n* = 2) and euthanized after 14 days (Supplemental Fig. 3A). Splenic T-cell responses were detected by IFN-γ ELISpot assay. Cells were stimulated with peptides spanning the entire GPC sequence of either the homologous Josiah strain (lineage IV) or one of three heterologous strains: Pinneo (lineage I), 803213 (lineage II), or GA391 (lineage III). No statistically significant differences were observed between the magnitudes of IFN-γ T-cell responses to homologous and heterologous LASV strains (mean ± SEM: Pinneo = 654.5 ± 189, 803213 = 755.9 ± 157, GA391 = 763.5 ± 96, Josiah = 1058 ± 159 SFU/10^6^ splenocytes) (Fig. 2a). Splenocytes isolated from animals receiving the control vaccine did not display reactivity to LASV GPC from any strain.

IgG antibody responses in mice vaccinated 28 days prior with ChAdOx1-Lassa-GPC (*n* = 8) or ChAdOx1-GFP (*n* = 2) were compared by ELISA (Supplemental Fig. 3B). Sera was incubated with GP antigen from the Josiah strain or one of two heterologous strains: LASV237 Nigeria 2010H (lineage II) or NIG08-A18 2008H (lineage III). Again, no statistically significant difference was observed between the titers of IgG antibodies recognizing homologous and heterologous LASV GP (mean ELISA titer: LASV237 Nigeria 2010H = 14000 ± 2832, NIG08-AI8 2008H = 13200 ± 2990, Josiah = 24800 ± 6219) (Fig. 2b). LASV GP-specific responses in the sera of ChAdOx1-GFP vaccinates fell at or below the limit of detection.

### ChAdOx1-Lassa-GPC vaccination induces a humoral response in guinea pigs

**Fig. 3:**
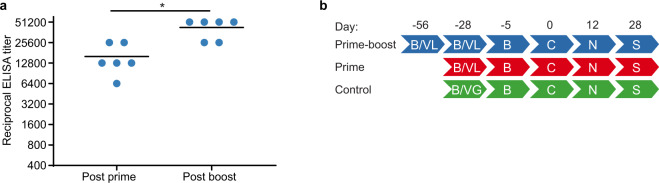
Vaccination of guinea pigs with ChAdOx1-Lassa-GPC induces a humoral immune response. **a** Serum samples were collected at 28 days post prime and post boost vaccination in the survival cohort (*n* = 6) of the prime-boost experimental group. LASV GP-specific IgG antibody titers in twofold serial-diluted sera were measured by ELISA. Horizontal lines indicate mean titers. Statistical significance between the post prime and post boost antibody titers was determined via Wilcoxon matched-pairs signed rank test (*p = 0.0312). **b** Summary of experimental timeline. VL = vaccination with ChAdOx1-Lassa-GPC (*n* = 10 per group); VG = vaccination with ChAdOx1-GFP (*n* = 10); B = blood collection; C = LASV challenge; N = planned necropsy for comparative virology (*n* = 4 per group); S = survival or study endpoint (after adjustment for loss of animals *n* = 5, *n* = 6, and *n* = 4 for the prime-boost, prime, and control experimental groups, respectively).

The protective efficacy of ChAdOx1-Lassa-GPC (after one or two doses) was evaluated in the Hartley guinea pig model of lethal Lassa fever. Three groups of animals (*n* = 10 per group) received the following immunizations: 3.0 × 10^8^ IU of ChAdOx1-Lassa-GPC on D-56 and D-28 (prime-boost), 3.0 × 10^8^ IU of ChAdOx1-Lassa-GPC on D-28 (prime), or 1.0 × 10^8^ IU of ChAdOx1-GFP on D-28 (control). The immunogenicity of ChAdOx1-Lassa-GPC in guinea pigs was confirmed by detection of GP-specific IgG antibodies 28 days post prime (D-28 mean ± SEM ELISA titer = 16000 ± 3200) and 28 days post boost vaccinations (D0 mean ± SEM ELISA titer = 42667 ± 5397) (Fig. 3a). A significant increase in IgG antibody titer was observed after administration of a second dose of ChAdOx1-Lassa-GPC (*p* = 0.0312, Wilcoxon matched-pairs signed rank test).

### ChAdOx1-Lassa-GPC vaccination does not induce a significant neutralizing antibody response against live guinea pig-adapted LASV or a LASV GP pseudotype

To assess the neutralizing capacity of antibodies produced after immunization with ChAdOx1-Lassa-GPC, sera collected on D0 from guinea pigs receiving the prime-boost vaccination regimen was incubated with guinea pig-adapted (GPA) LASV prior to the inoculation of Vero E6 cells. Sera from the prime-boost vaccination group was assayed as these animals developed the highest virus-specific IgG titers as detected by ELISA. After 10 days, cytopathic effect (CPE) was observed in all samples at the lowest dilution (1:10). Therefore, all sera were considered negative and neutralizing titers were reported as falling below the minimum dilution assayed (Supplemental Table 1). A cocktail of human monoclonal antibodies (25.10C, 12.1F, 37.2D, and 8.9F) served as a positive control and demonstrated inhibition at a total antibody concentration of 15 µg/mL, while CPE was observed at 1.5 µg/mL.

To verify results, the above sera samples were also tested for their capacity to neutralize a replication-deficient, GFP-expressing vesicular stomatitis virus (VSV) pseudotyped with Josiah strain LASV GP, VSV-Lassa-GPc-cFLAG. Sera was incubated with VSV-Lassa-GPc-cFLAG for 1 h prior to infection of Vero E6 cells. After 16 h, GFP-positive cells were counted and the proportion of infected cells relative to a virus only control was determined. Little to no inhibition of virus infection was observed at the lowest sera dilution (1:10); neutralizing titers were reported as half-maximal inhibition (IC_50_) values (Supplemental Table 1). Assay validation was performed using the four positive control human monoclonal antibodies described above, both individually and in a cocktail at a starting concentration of 30 µg/mL. Neutralizing activity was observed at similar concentrations as previously described^45^.

### Immunization with ChAdOx1-Lassa-GPC confers full protection against lethal disease

During the course of the study, prior to challenge, three animals died for reasons unrelated to vaccination or LASV challenge, altering the total number of animals in the ChAdOx1-Lassa-GPC prime-boost vaccination (*n* = 9) and control (*n* = 8) experimental groups. To account for this reduction, the numbers of animals in the survival cohorts of the prime-boost (*n* = 5) and control (*n* = 4) groups were adjusted accordingly.

On D0, all animals were challenged with 1.0 × 10^5^ TCID_50_ of GPA-Josiah strain LASV, which was passaged four times in Hartley guinea pigs as previously described^38^. GPA LASV harbors a single nucleotide polymorphism in the S genomic segment compared to the wild-type Josiah strain virus.

**Fig. 4:**
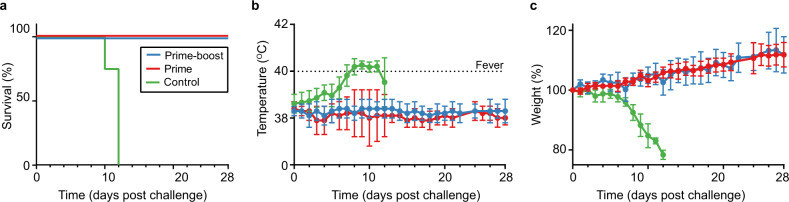
ChAdOx1-Lassa-GPC-vaccinated guinea pigs are protected against lethal LASV challenge. **a** Survival of Hartley guinea pigs challenged with GPA LASV. Survival of vaccinated animals was significant compared to control animals by Mantel–Cox log-rank test (***p = 0.0005). **b** Mean temperature of challenged animals; animals with a temperature of ≥40 °C were diagnosed as febrile. **c** Mean weight loss post LASV inoculation expressed as a percentage of animals’ weight at the time of challenge (D0). Error bars represent the standard deviation of the mean for the survival cohort.

On D12 post challenge, four randomly selected animals from each group were euthanized to perform virological assessments in lung, liver, spleen, and sera. The remaining animals were used to assess survival; animals in the survival cohort were euthanized after meeting humane endpoint criteria or on D-28 post challenge, which marked the study endpoint (Fig. 3b). All survival cohort guinea pigs vaccinated with ChAdOx1-Lassa-GPC survived challenge and did not develop fevers, experience weight loss, or exhibit other signs of disease (Fig. 4a). No discernable differences in weight or temperature were observed between prime and prime-boost vaccinates. By contrast, all control animals in the survival cohort developed signs of terminal illness and met humane endpoint criteria (>20% weight loss) on or before D12. The temperatures of control animals began to increase on D3 and animals became febrile by D8. Weight loss was observed by D7 and progressed until euthanasia by D12 (Fig. 4b, c).

### Prime-boost vaccination reduces levels of replicating LASV in tissues after challenge

**Fig. 5:**
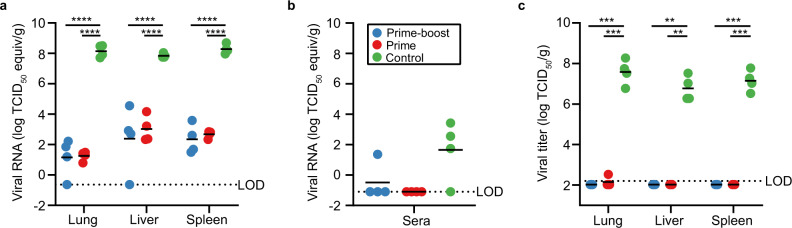
Viral load is significantly lower in the tissues and sera of ChAdOx1-Lassa-GPC vaccinates compared to controls. Lung, liver, spleen, and sera samples were collected from animals (*n* = 4/group) during post-challenge necropsy (D12). Detection of LASV RNA in the tissues (**a**) and sera (**b**) of vaccinated and control animals by qRT-PCR. The dotted lines represent the limits of detection (LOD) for analysis of tissue (−0.64 log TCID_50_/g equivalents) and sera (−1.10 log TCID_50_/mL equivalents) due to distinct inactivation methods used to remove these samples from BSL4 containment. Differences in viral load in the lung, liver, and spleen of vaccinated animals versus controls were statistically significant by two-way ANOVA with Tukey’s multiple comparisons test (****p < 0.0001). Levels of LASV RNA detected in sera did not differ significantly between groups by Kruskal–Wallis nonparametric test with Dunn’s multiple comparisons. **c** Infectious virus titers in tissues positive for LASV RNA (lung, liver, and spleen) were determined via endpoint titration on Vero E6 cells. The dotted line represents the LOD (2.20 log TCID_50_/g). Differences in virus titers between vaccinated and control animals were statistically significant in all tissues by two-way ANOVA with Tukey’s multiple comparisons test (**p = 0.0012, ***p = 0.0008, 0.0006, 0.0002).

Despite conferring 100% protection against clinical disease after LASV challenge, vaccination with ChAdOx1-Lassa-GPC did not induce sterile immunity. Low amounts of LASV RNA were detected in the tissues of immunized animals necropsied on D12 by qRT-PCR (Fig. 5a). In the lungs, mean differences (±SEM) in viral load between the control animals and prime-boost or prime vaccinates were 6.99 ± 0.68 and 6.90 ± 0.68 log TCID_50_/g equivalents, respectively. Similarly, mean viral loads in the livers of control animals exceeded that of animals in the prime-boost and prime groups by 5.46 ± 0.68 and 4.83 ± 0.68 log TCID_50_/g equivalents, respectively. Finally, vaccination with ChAdOx1-Lassa-GPC reduced splenic viral load by 5.94 ± 0.68 and 5.61 ± 0.68 log TCID_50_/g equivalents after prime-boost or single-dose delivery, respectively. Reductions in viral RNA in the vaccinated animals versus controls were statistically significant for all tissues (*p* < 0.0001, two-way ANOVA with Tukey’s multiple comparisons test). Meanwhile, no significant differences were observed between the prime-boost and prime immunization groups.

Overall, viral loads were much lower in sera than in the tissues (Fig. 5b). On D12, qRT-PCR analysis did not detect LASV RNA in the sera of ChAdOx1-Lassa-GPC vaccinates, apart from one animal in the prime-boost group (1.36 log TCID_50_/mL equivalents). By contrast, sera from all but one control animal was positive for LASV RNA (mean ± SEM: 1.66 ± 0.98 TCID_50_/mL equivalents). Statistically significant differences in mean serum viral load were not observed between vaccinated and control animals.

Controls designated to the survival cohort met humane endpoint criteria at nearly the same time as the scheduled necropsy for virological assessment (*n* = 4 per group); as such, similarly high viral loads were measured in all of the control terminal samples. Viral RNA was still detected in the terminal tissues and sera samples of some survival cohort vaccinates at the study endpoint (D-28), but levels had decreased (Supplemental Fig. 4A, 4B).

Antibody responses specific to LASV nucleoprotein (NP) were mounted in vaccinated and control animals by D12, which further suggested that low-level virus replication occurred (mean ± SEM ELISA titer: prime-boost = 1500 ± 619, prime = 24000 ± 9906, control = 20800 ± 4800). The titer of anti-NP IgG antibodies was significantly lower in animals receiving prime-boost vaccination compared to those receiving a single dose or control vaccination (*p* = 0.0492, Kruskal–Wallis nonparametric test with Dunn’s multiple comparisons) (Supplemental Fig. 5).

To determine if LASV RNA detected by qRT-PCR was indicative of infectious virus, we titrated lung, liver, and spleen tissues collected on D12 on Vero E6 cells (Fig. 5C). No viable LASV was isolated from the tissues of any of the animals immunized with ChAdOx1-Lassa-GPC, besides a single lung sample from an animal in the prime vaccination group (2.52 log TCID_50_/g) at just above the limit of detection of the assay. Meanwhile, infectious virus was detected in all tissues isolated from control animals (mean ± SEM in lung, liver, spleen: 7.58 ± 0.31, 6.77 ± 0.31, 7.15 ± 0.26 log TCID_50_/g). Differences in mean virus titer between prime-boost vaccinates and controls (lung, liver, spleen: *p* = 0.0008, 0.0012, 0.0006), as well as between prime vaccinates and controls (lung, liver, spleen: *p* = 0.0002, 0.0012, 0.0006) were statistically significant (two-way ANOVA with Tukey’s multiple comparisons test).

**Fig. 6:**
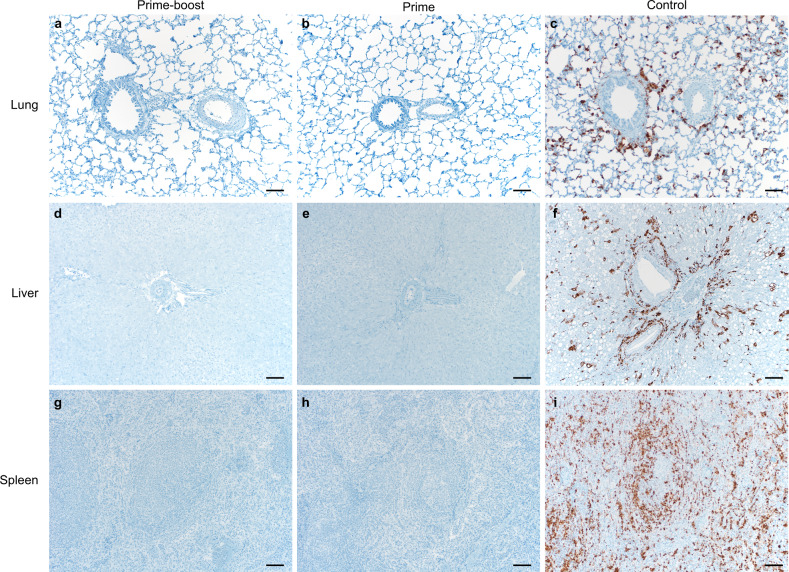
Replicating LASV is not detectable in tissues of ChAdOx1-Lassa-GPC-vaccinated animals by in situ hybridization. Lung, liver, and spleen samples were collected from guinea pigs (*n* = 4/group) during post-challenge necropsy (D12). Tissue sections were stained with a LASV RNA-specific probe. Representative fields of view for each tissue collected from the prime-boost (**a**, **d**, **g**), prime (**b**, **e**, **h**), and control groups (**c**, **f**, **i**) were taken at ×100 magnification. The scale bar represents 50 µm. Positive-strand RNA, indicative of virus replication, is visible as brown staining.

Lung, liver, and spleen tissue sections were stained with hematoxylin and eosin or with LASV-specific in situ hybridization (ISH) probes to detect replicating virus (Fig. 6). All slides were evaluated by a board-certified veterinary pathologist blinded to study group allocations. Limited pathology was observed in the hematoxylin and eosin stained tissues of vaccinated animals euthanized on D12 post challenge. Marked and severe signs of vacuolar degeneration and centrilobular-midzonal bridging were detected in the livers of one and three control animals, respectively, but not in the vaccinated animals. Vacuolar degeneration is a response to stress or damage to liver cells, which may be caused by disease processes, including but not limited to, infection, stress, and inflammation. Finally, the lung tissue in three of the four control animals exhibited mild-to-marked interstitial pneumonia with macrocytosis, while the lungs of vaccinates were free of this disease phenotype. ISH staining was largely absent in animals vaccinated with ChAdOx1-Lassa-GPC (Fig. 6). However, a single lung sample from the prime-boost vaccination group exhibited low-level ISH staining for positive-strand LASV RNA. By contrast, LASV RNA staining was abundantly present in all investigated tissues of control animals. Specifically, LASV replication was observed in type I and II pneumocytes and alveolar macrophages (Fig. 6c). LASV replication was also observed in hepatic Kupffer cells (Fig. 6f) and splenic reticuloendothelial cells (Fig. 6i).

## Discussion

Lassa fever is a public health threat requiring continued research momentum due to its potential to cause significant morbidity and mortality. Disease prevention efforts would be enhanced by the availability of a safe and efficacious vaccine. The WHO Target Product Profile (TPP) outlines preferred criteria for a LASV vaccine candidate deployed in a non-emergency setting. These include the following: safety across age groups and in pregnant women, efficacy after a single dose, durable protection, conferral of heterosubtypic immunity, thermostability, and ease of large-scale manufacturing for stockpiling^46^. Here, we reported results from preclinical immunogenicity and efficacy evaluations of a chimpanzee adenovirus-vectored Lassa fever vaccine candidate, ChAdOx1-Lassa-GPC. Our data suggest that ChAdOx1-Lassa-GPC is a promising LASV vaccine candidate and warrants further studies to determine if it will meet the WHO TPP criteria. ChAdOx1 is replication-deficient, contributing to low reactogenicity and an enhanced safety profile, which has been demonstrated in clinical trials’ subjects ranging from 5 months to over 70 years of age^47–52^. Despite producing only a single-cycle infection, ChAdOx1 is highly immunogenic, achieving persistent antigen expression and is a potent inducer of cellular and humoral immune responses. ChAdOx1-vectored vaccines are also thermostable up to 45 °C without significant loss of potency, eliminating the need for uninterrupted cold-chain access^53^. Most significantly, ChAdOx1-Lassa-GPC demonstrated 100% efficacy in a guinea pig model of Lassa fever. Hartley guinea pigs receiving a single dose of ChAdOx1-Lassa-GPC were fully protected against lethal LASV challenge and did not develop observable signs of illness, in contrast to control vaccinated animals. At D12 post challenge, no viable virus was recovered from animals receiving two doses of ChAdOx1-Lassa-GPC. Only one animal receiving a single dose of the vaccine had recoverable infectious virus in its lung tissue, at just over the limit of detection for the assay. However, the observation of low levels of LASV RNA in the tissues of vaccinates indicated that sterilizing immunity was not achieved. Induction of a nucleoprotein-specific antibody response in vaccinated (and control) animals provided further evidence of virus replication; however, the titers in prime-boost vaccinates were significantly lower than those in animals receiving a single dose of ChAdOx1-Lassa-GPC or in control animals. Together, these findings suggest that while administration of a booster vaccination is not necessary to protect against LASV-associated disease in guinea pigs, it does reduce viral load in the days after infection.

It is unclear if the observation of replicating virus in immunized animals is predictive of outcomes in non-human primates or reflective of the stringency of this particular challenge model. Few vaccine candidates have been tested in Hartley guinea pigs against lethal GPA-Josiah LASV challenge. Instead, most rodent efficacy studies have relied on the inbred strain 13 guinea pig model. Multiple vaccine candidates, including leading candidates based on the vesicular stomatitis virus (VSV) platform and the attenuated Mopeia virus (MOPV) reassortant clone ML29, have reportedly conferred sterilizing protection in these animals^54,55^. However, the same VSV-vectored vaccine candidate demonstrated only 83% efficacy in Hartley guinea pigs and infectious virus was isolated from the liver, lung, and spleen of vaccinated animals^56^. These findings may suggest that the Hartley guinea pig model represents a more stringent model of LASV infection, potentially making it more likely to observe residual virus after challenge. Evaluation in non-human primates may provide a clearer indication of a vaccine’s ability to completely protect after lethal LASV challenge. Non-human primate vaccine efficacy studies for ML29, VSV-vectored, MV-vectored, and DNA vaccine candidates have documented transient viremia in some or all animals after LASV challenge, which appeared around 6–7 days post challenge and was no longer detectable within a few days^24,26,27,32^. ML29 and MV-vectored vaccine efficacy studies in marmosets and cynomolgus macaques (respectively) reported that the tissues of vaccinated animals were negative for infectious LASV according to plaque titration assays, indicating complete viral clearance by the time of necropsy or study endpoint^26,32^. Combined with observations of transient viremia, these findings indicate that these vaccines achieved nearly sterilizing immunity in non-human primates.

The correlates of protection for Lassa fever are poorly understood. It is widely hypothesized that a strong cellular immune response is necessary to protect against natural infection. This assertion is predicated on evidence that a strong humoral response is not observed in patients during the acute phase of the disease and neutralizing antibodies are not detected until patients are well into convalescence^39^. In addition, in non-human primates, a strong T-cell response is observed in animals that successfully control infection, while a lack of T-cell activation is observed in animals developing fatal disease^57^. Furthermore, in humans, LASV-specific CD4^+^ and CD8^+^ T-cell responses are activated early during infection and continue to be detected after recovery despite low (or absent) antibody responses^58^. Vaccine development for Lassa fever would be enhanced by further studies into vaccine-mediated cellular immune mechanisms. However, tools for these investigations are not well-developed in the guinea pig model. For this reason, we carried out immunogenicity experiments in mice, to assess the quality of the cellular immune response after vaccination with ChAdOx1-Lassa-GPC. Immunized mice developed a strong LASV-specific CD8^+^ T-cell response. The majority of antigen-experienced CD8^+^ T cells secreted IFN-γ and TNF-α. IL-2 expression was low overall. Evaluations of VSV-vectored and MV-vectored Lassa fever vaccines have similarly observed an IFN-γ- and TNF-α-driven effector response post vaccination; IL-2 expression in response to antigen re-exposure was not assessed in these studies^27,32^.

There is ample evidence suggesting that multiple-cytokine-producing T cells are functionally superior to single-cytokine-producing T cells, as the latter have a limited capacity to be sustained as memory cells^59^. Hence, vaccines, such as ChAdOx1-Lassa-GPC, which elicit a high proportion of polyfunctional T cells may be more likely to confer long-term protection. Studies characterizing vaccine-induced T-cell responses against HIV, HCV, and influenza, revealed a strong association between the level of protection and the induction of high frequencies of T cells co-producing IFN-γ, TNF-α, or IL-2^59–63^. At 3 weeks post immunization, the vaccine-specific CD8^+^ T-cell response was skewed toward effector or effector memory function. Data from other adenoviral-vectored vaccine candidates suggest that the central memory response is detectable only from a few months after vaccination. These kinetics may have contributed to low frequency IL-2 expression upon antigenic stimulation of splenocytes collected 21 days after immunization, as central memory T cells are significant producers of this cytokine^59^. Administration of a second dose of ChAdOx1-Lassa-GPC favored expansion of effector cells but did not significantly alter the overall proportion of activated cells or their functionality. This finding was not unexpected as administration of a heterologous viral vector prime-boost vaccination regimen may be required to efficiently boost and expand polyfunctional cellular immune responses^49,50,52^.

Recently, a report on the efficacy of several MV-vectored Lassa fever vaccines included detailed transcriptomic profiling of innate and cellular immune responses after vaccination^32^. The intensity of GP-specific CD8^+^ (and to a lesser extent CD4^+^) T-cell responses appeared to be associated with low clinical scores and viremia. Furthermore, efficient control of LASV after immunization was associated with cytotoxic T-cell activation. Ultimately, clear indicators of correlates of protection for LASV could not be gleaned from the data. Nevertheless, this study offered detailed transcriptional and proteomic profiling of the post-vaccination response, which has not been previously supplied for a LASV vaccine^32^. Such studies may yield important insight into the complex effector mechanisms underlying vaccine-mediated cellular immunity to LASV infection, which may eventually lead to the definition of correlates of protection and improved vaccine design.

A single dose of ChAdOx1-Lassa-GPC produced a humoral response, which was boosted by administration of a second dose. However, the observed increase in mean IgG titers was only statistically significant in guinea pigs and not in mice. These antibodies did not demonstrate neutralizing activity in live virus or pseudotype-based assays. The functional role and relative importance of antibodies in controlling LASV infection has not been well-characterized. During natural infection, antibody production is thwarted due to viral targeting of antigen-presenting cells; antibodies, if present, may confer some degree of protection, as demonstrated by successful monoclonal antibody therapy in guinea pigs and non-human primates^64,65^. Neutralizing antibodies to LASV GP have been detected in some survivors of LASV infection and primarily target epitopes exposed in the prefusion trimer. However, it is postulated that the glycan arrangement around key neutralizing sites in the prefusion antigen also precludes the formation of high-affinity neutralizing antibodies^66^. In a recent study, adjuvanted virus-like particles expressing a stable native-like LASV Josiah GP trimer induced the production of hyperimmune sera in rabbits with the capacity to neutralize multiple strains of LASV in vitro. It has yet to be determined if this system can induce sufficiently high titers of neutralizing antibodies to protect against LASV challenge in animal models^67^. Meanwhile, another study has suggested that non-neutralizing antibodies may play a role in controlling infection through antibody-dependent cellular phagocytosis or antibody-dependent cellular cytotoxicity^29^. Again, assays to investigate these mechanisms in guinea pigs are largely not available, but there is potential for functional studies to be performed in mice and non-human primates.

As outlined in the WHO TPP, heterosubtypic immunity is a key requirement for LASV vaccines. The antigens included in most vaccine candidates and diagnostic tools for Lassa fever have been developed against the canonical lineage IV Josiah strain, originally isolated and characterized in Sierra Leone in 1976. However, there are at least seven different lineages of LASV with significant genetic diversity between them. We tested the cross-reactivity of IFN-γ T cells and serum IgG antibodies in ChAdOx1-Lassa-GPC vaccinates to GP from different LASV strains. We selected representative strains from lineages I, II, and III for ELISpot analysis and strains from lineages II and III for ELISA analysis, and compared heterologous responses to the homologous Josiah strain GP. Lineages II and III are most epidemiologically relevant in Nigeria, while lineage IV circulates in Sierra Leone, Liberia, and Guinea^2,21^. An insufficient number of isolates or cases in lineages V, VI, and VII makes it difficult to assess their contribution to current caseloads or outbreaks^5,22,23^. Our results indicate that although the responses to the homologous antigen were highest, the apparent decrease in T-cell and antibody responses to the heterologous antigens were not statistically significant. Therefore, we expect ChAdOx1-Lassa-GPC will confer at least some degree of protection against a diverse array of LASV strains.

Our assessment of ChAdOx1-Lassa-GPC comprised data from two preclinical models: CD-1 mice and Hartley guinea pigs. Due to the paucity of tools to facilitate immunological assays in guinea pigs, immunogenicity evaluations were completed in mice. However, as mice are not susceptible to Lassa fever, it was not possible to extend the use of this model to efficacy evaluation. As previously mentioned, the impact of administering a second dose of ChAdOx1-Lassa-GPC differed between mice and guinea pigs. Mice receiving prime-boost immunization with ChAdOx1-Lassa-GPC developed a cellular immune response of comparable magnitude and quality compared to mice receiving a single dose of the vaccine, but this was characterized by a more dominant effector response. The humoral response, as measured by IgG ELISA, showed a trend toward enhancement in prime-boost animals, but this was not statistically significant. By contrast, in Hartley guinea pigs, IgG titers were significantly boosted after delivery of a second dose of the vaccine. Furthermore, prime-boost animals exhibited reduced viral load compared to prime only animals after infection. We did not find these differences in results to be implausible given the previously discussed variation in outcomes observed after LASV vaccination and challenge across animal models. Combined vaccine immunogenicity and efficacy evaluation in a single non-human primate species will likely provide the most useful evidence toward predicting how ChAdOx1-Lassa-GPC will perform in humans.

Collectively, our findings demonstrate that ChAdOx1-Lassa-GPC is effective in protecting Hartley guinea pigs against a uniformly lethal homologous LASV challenge. The results lay the foundation for future work to assess the vaccine’s capacity to protect against infection by virus strains of other LASV lineages in a heterologous challenge study. In addition, they justify progression to expanded immunogenicity characterization and efficacy determination in a non-human primate model.

## Methods

### Animal studies ethics statement

All procedures in CD-1 mice were performed as permitted by UK Home Office Project License P9804B4F1 and conducted in accordance with the Animal (Scientific Procedures) Act 1986 with approval by the University of Oxford Animal Care and Ethical Review Committee. LASV challenge experiments in Hartley guinea pigs were approved by the Institutional Animal Care and Use Committee (ACUC) at Rocky Mountain Laboratories (RML). Experiments were performed in an Association for Assessment and Accreditation of Laboratory Animal Care (AAALAC) approved facility, following the guidelines and basic principles in the NIH Guide for the Care and Use of Laboratory Animals, the Animal Welfare Act (United States Department of Agriculture), and the United States Public Health Service Policy on Humane Care and Use of Laboratory Animals. BSL4 protocols, sample inactivation protocols, and standard operating procedures for removal of specimens from high containment were approved by the Institutional Biosafety Committee (IBC).

### Cells and virus

The glycoprotein precursor (GPC) gene from Josiah strain LASV (GenBank accession number J04324.1, position 1872-3374) was codon-optimized for humans and synthesized by GeneArt (Thermo Fisher Scientific). The synthesized G gene was cloned into a transgene expression plasmid comprising a modified human cytomegalovirus immediate early promoter (CMV promoter) with tetracycline operator (TetO) sites and the polyadenylation signal from bovine growth hormone (BGH). The resulting expression cassette was inserted into the E1 locus of a genomic clone of ChAdOx1 using site-specific recombination^47^. The virus was rescued and propagated in T-REx-293 cells (Invitrogen). Purification was by CsCl gradient ultracentrifugation, and the virus was titered as previously described^68^. Doses for vaccination were based on infectious units (IU).

### Immunogenicity experiments in mice

Female CD-1 mice (Charles River Ltd, Harlow, UK), aged 6–8 weeks, were immunized via intramuscular injection in the left hind leg. A dose of 1.0 × 10^8^ IU of the appropriate vaccine was delivered in a total volume of 50 μL and diluted in sterile PBS. Where applicable, prime and boost vaccinations were administered 4 weeks apart. Study endpoint was either 21 days (single dose versus prime-boost vaccination experiment), 14 days (investigation of heterosubtypic cellular immunity), or 28 days (evaluation of cross-reactive antibody responses) after immunization. At the study endpoint, blood was collected via cardiac puncture, animals were euthanized via cervical dislocation, and spleens were harvested for further immunological analysis.

### ELISpot, surface and intracellular cytokine staining, and flow cytometry

Splenocytes were isolated for analysis via IFN-γ ELISpot, surface and intracellular cytokine staining (ICS) and flow cytometry as previously described^69–71^. Splenocytes were restimulated with 2 μg/mL of the appropriate antigenic peptide pool (comprised of individual peptides that were 20 amino acids in length with 10 amino acids of overlap) spanning the full-length LASV GPC sequence (Mimotopes). Reference sequences for peptide synthesis are as follows: NP_694870.1 (Josiah), AIT17836.1 (Pinneo), AAF86703.1 (803213), CAA36645.1 (GA391). Cells were restimulated for 18–20 h or 6 h (at 37 °C and 5% CO_2_) for IFN-γ ELISpot and ICS, respectively. Surface staining and ICS were carried out using the following antibodies: LIVE/DEAD® Fixable Aqua Dead Cell Stain Kit (Thermo Fischer Scientific); anti-CD8a-PerCP/Cy5.5, anti-CD62L-PeCy7, anti-IFN-γ-eFluor 450, anti-TNF-α-Alexa Fluor 488, anti-IL-2-PE (eBioscience); anti-CD4-Brilliant Violet 650, anti-CD44-Alexa Fluor 700 (BioLegend); and anti-CD127-eFluor 660 (Invitrogen). Antigen-specific T-cell responses were quantified by subtracting the response (SFU for IFN-γ ELISpot and the percentage of cytokine-positive cells for ICS) measured without stimulation from that observed after restimulation.

### ELISA

Endpoint titer ELISA was performed as previously described^72^. Murine ELISA analysis was carried out using 2 μg/mL of the relevant LASV GP coating antigen (Zalgen Labs, LLC). Goat anti-mouse whole IgG conjugated to alkaline phosphatase and 20 mg *p*-nitrophenylphosphate detection substrate were used (Sigma). ELISAs were performed on irradiated guinea pig sera using ReLASV® Pan-Lassa IgG/IgM ELISA Test Kit (Zalgen Labs, LLC) with either GP or NP as the capture antigen. Tests were performed according to the manufacturer’s instructions with the modification of using goat anti-guinea pig whole IgG conjugated to HRP (SeraCare) diluted 1:1000 in the diluent provided in the kit as secondary antibody. All sera samples were prepared in a twofold serial dilution starting at a 1:400 dilution in PBS with 0.05% tween-20 (mouse) or kit diluent (guinea pig). The antibody levels were normalized to naive CD-1 mouse or guinea pig sera. A limit of detection of three times the standard deviation of the mean OD value for naive sera was used to determine the endpoint titer.

### Vaccination and LASV challenge of Hartley guinea pigs

Mixed gender Hartley strain guinea pigs (*Cavia porcellus*), age matched to 14 weeks at the time of challenge, were vaccinated intramuscularly with 3.0 × 10^8^ IU of ChAdOx1-Lassa-GPC or 1.0 × 10^8^ IU of ChAdOx1-GFP (control vaccine). The prime-boost group (*n* = 10) were immunized with ChAdOx1-Lassa-GPC on D-56 and D-28, the prime group (*n* = 10) received a single dose of ChAdOx1-Lassa-GPC on D-28, and control animals (*n* = 10) received a single dose of ChAdOx1-GFP vaccine on D-28. On D0, animals were challenged with 1.0 × 10^5^ plaque-forming units of Josiah strain-derived GPA LASV (Genbank accession numbers MW004546 (L segment) and MW004547 (S segment)), which is equivalent to 100 times 50% of the lethal dose (LD_50_) via two intraperitoneal injections^38^. Animals underwent daily post-challenge monitoring for body temperature, weight, and signs of disease (lethargy, guarded posture, and labored respiration). At D12 post challenge, four animals from each group were euthanized; blood, lung, liver, and spleen samples collected for virological and histopathological analyses. The remaining animals in each group comprised the survival cohorts; these animals were euthanized on D-28 (study endpoint), unless they met humane endpoint criteria earlier. Blood, lung, liver, and spleen samples were collected for virological and histopathological analyses.

### Virus neutralization assay with live GPA LASV

The virus neutralization assay was carried out on Vero E6 cells in a 96-well plate format under biosafety level 4 conditions. Sera was complement inactivated at 56 °C for 30 min and diluted twofold in a series beginning from 1:10. A cocktail of human monoclonal antibodies—25.10C, 12.1F, 37.2D, and 8.9F (Zalgen Labs, LLC)—served as the positive control and was ten-fold serially diluted starting from a (total antibody) concentration of 15 μg/mL. Naive guinea pig serum and media only wells were used as negative controls. The diluted sera or antibody cocktail was incubated with 20 TCID_50_ of GPA LASV at 37 °C for 1 h. The cell culture supernatant was removed and 100 µL of the above mixture was added to the cells. Infected cell cultures were incubated at 37 °C, 5% CO_2_ and scored for cytopathic effect (CPE) after ten days. Virus neutralizing titers were reported as the maximum reciprocal sera dilution at which CPE was absent or as <10 in the case of a negative result.

### Pseudotype-based virus neutralization assay

VSV pseudotyped with LASV GP, VSV-Lassa-GPc-cFLAG, was used as an additional model for the assessment of neutralization under biosafety level 2 conditions. To generate Lassa-GPc-cFLAG, the sequence for LASV GPC Josiah strain was codon-optimized for human cells and further modified by addition of a 3′ tri-glycine linker (GGGS) and the nucleotide sequence for a FLAG-tag (DYKDDDDK). Lassa-GPc-cFLAG was synthesized and cloned into pcDNA3.1+ (GenScript); accuracy of the construct was confirmed by Sanger sequencing (ACGT). Replication-incompetent pseudotyped VSV particles expressing GFP and luciferase reporters were produced as previously described^73–75^.

Sera was gamma-irradiated prior to transfer from biosafety level 4 to biosafety level 2 conditions, complement inactivated at 56 °C for 30 min, and serially diluted (by a factor of 2.5) from 1:10 to 1:2441. Five-fold serial dilutions of human monoclonal antibodies 25.10C, 12.1F, 37.2D, and 8.9F from a starting concentration of 60 µg/mL served as positive controls. A cocktail of all four antibodies, at an initial (total antibody) concentration of 60 µg/mL, was serially diluted in the same way as an additional positive control. Naive guinea pig serum and virus only wells were used as negative controls. Subsequently, 1000 FFU of VSV-Lassa-GPc-cFLAG were added to the diluted sera or antibody and incubated for 1 h at 37 °C and 5% CO_2_. Virus-antibody mixtures were added to confluent Vero E6 cells and spin infection was carried out at 4 °C at 1200 × *g* for 1 h. Infected cells were cultured for 16 h. GFP-positive cells were counted using the ImmunoSpot® 7 Software and CTL Analyzer (CTL Analyzers, LLC). The percent of infected cells was determined relative to uninhibited virus only control wells. Half-maximal inhibition (IC_50_) values were calculated based on reciprocal sera dilution by GraphPad® Prism 8 using a sigmoidal nonlinear fit model (4PL regression curve). Values above 100% infectivity were converted to 100%.

### Real-time quantitative reverse-transcriptase PCR analysis

Whole blood samples were collected from guinea pigs on D12 and D-28. Blood was collected from the superior vena cava or, in the case of terminal bleeds, via cardiac puncture under anesthesia. Virus was inactivated and RNA extracted following approved institutional protocols. Briefly, 140 µL of blood was added to 560 mL of AVL (Qiagen) and incubated at room temperature (RT) for 10 min; this mixture was then added to 560 µL of absolute ethanol. RNA was extracted using a QIAamp Viral RNA Mini Kit (Qiagen) according to the manufacturer’s instructions. Tissues were weighed, homogenized in RLT buffer (Qiagen), and adjusted to a concentration of 30 mg of tissue in 600 µL of RLT. The homogenate was incubated at RT for 10 min and subsequently added to 600 µL of 70% ethanol. RNA was then extracted using a RNeasy Mini Kit (Qiagen) following the manufacturer’s instructions.

Presence of LASV RNA was detected using real-time quantitative reverse-transcriptase PCR (qRT-PCR). qRT-PCR assays were performed using 5 µL of template in 20 µL QuantiFast master mix (Qiagen) on a Qiagen Rotor-Gene instrument. Forward and reverse primers LaV F2 CCACCATYTTRTGCATRTGCCA and LaV R GCACATGTNTCHTAYAGYATGGAYCA, respectively, and probe LaV TM AARTGGGGYCCDATGATGTGYCCWTT with a 5′ FAM were used^76^. The standard curve was generated from a 10-fold dilution of RNA extracted from stock GPA LASV with a known virus concentration.

### Virus titration assay

Endpoint titrations were carried out on Vero E6 cells inoculated with ten-fold dilutions of serum or tissue homogenates in a 96-well plate format. The titration plates were incubated for 1 h at 37 °C and 5% CO_2_. After incubation, the diluted sera or tissue solutions were removed, and the plates were rinsed twice with sterile PBS. Finally, 200 µL of DMEM with 2% BSA was added to each well; plates were incubated at 37 °C, 5% CO_2_ and scored for cytopathic effect after 10 days. TCID_50_ values were calculated using four replicates according to the Spearman-Karber method^77^.

### Histology and in situ hybridization

Liver, spleen, and lung samples were placed into paraffin-embedding cassettes and formalin fixed for 7 days. Whole lungs were formalin fixed for 30 days. Paraffin-embedded tissues were thinly sliced and probed for viral RNA by in situ hybridization (ISH). ISH was performed using the RNAscope 2.5 VS assay (Advanced Cell Diagnostics, Inc., Newark, CA) targeting Josiah strain Lassa virus (GenBank accession number J04324.1, position 355-1782) positive sense mRNA on the Ventana Discovery ULTRA slide auto staining system (Ventana Medical Systems Inc., Tucson, USA). A board-certified veterinary anatomic pathologist blindly evaluated all tissue slides.

### Statistical analysis

Statistical analyses were performed with Prism software version 7.04 and 8.0 (GraphPad) using the specific tests noted within the text.

### Reporting summary

Further information on experimental design is available in the Nature Research Reporting Summary linked to this paper.

## Supplementary information

Supplementary information

Reporting Summary

## Data Availability

Data will be deposited in Figshare: 10.6084/m9.figshare.12425165.v1.
