# Supplementary material for: ChAdOx1-vectored Lassa fever vaccine elicits a robust cellular and humoral immune response and protects guinea pigs against lethal Lassa virus challenge
Source: NPJ Vaccines. 2021 Mar 2;6:32. doi: 10.1038/s41541-021-00291-x (PMC7925663; doi:10.1038/s41541-021-00291-x)
Supplement: Supplementary file 1 — Supplementary information [file 41541_2021_291_MOESM1_ESM.pdf]

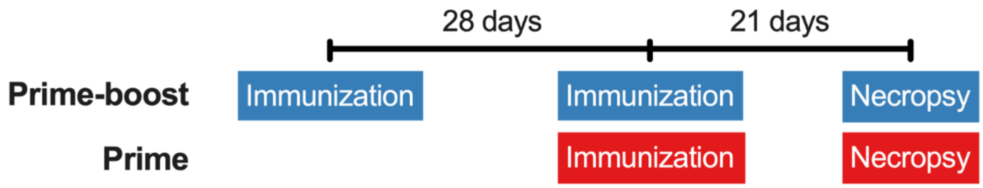

Supplemental Fig. 1. Murine cellular immunogenicity experimental timeline. Mice were immunized with 1.0×10^8 IU of ChAdOx1-Lassa-GPC in a single dose (n=8) or prime-boost (n=8) regimen. Prime and boost immunizations were performed 28 days apart. All animals were necropsied 21 days after final immunization and spleen and serum samples were collected for analysis.

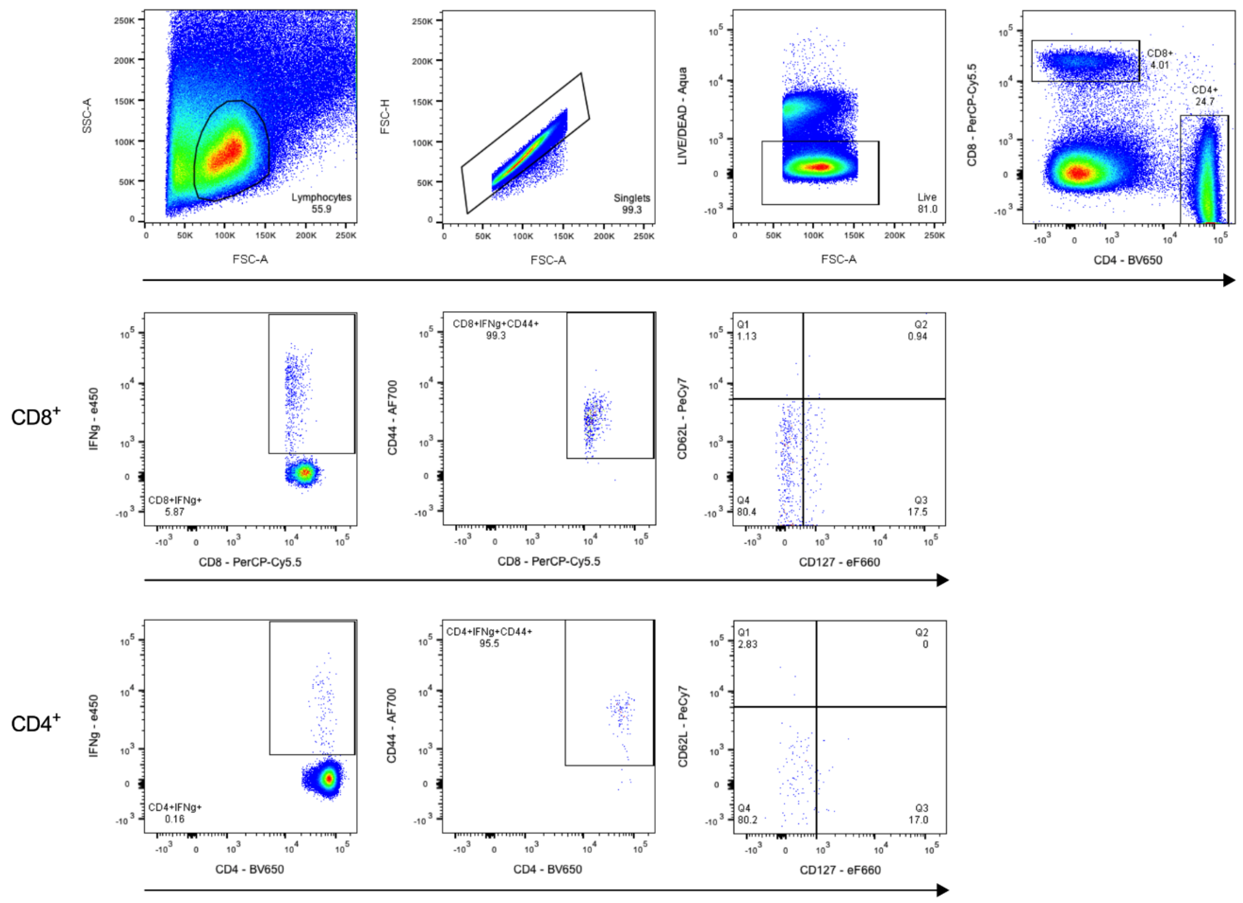

Supplemental Fig. 2. Flow cytometry data gating strategy. All events were first depicted using a combination of forward scatter area (FSC-A) and side scatter area (SSC-A). Lymphocytes were gated and displayed using a combination of FSC-A and forward scatter height (FSC-H), singlets were isolated, and live cells were gated using LIVE/DEAD and FSC-A. $CD8^+$ and $CD4^+$ T cell gates are shown. LASV-specific cells were identified as IFN-γ^+. All memory subsets were distinguished by first gating $CD44^+$ cells; effector cells (T_E) were identified as $CD62L^-$ $CD127^-$, effector memory cells (T_{EM}) were identified as $CD62L^-$ $CD127^+$, and central memory cells (T_{CM}) were identified as $CD62L^+$ $CD127^+$.

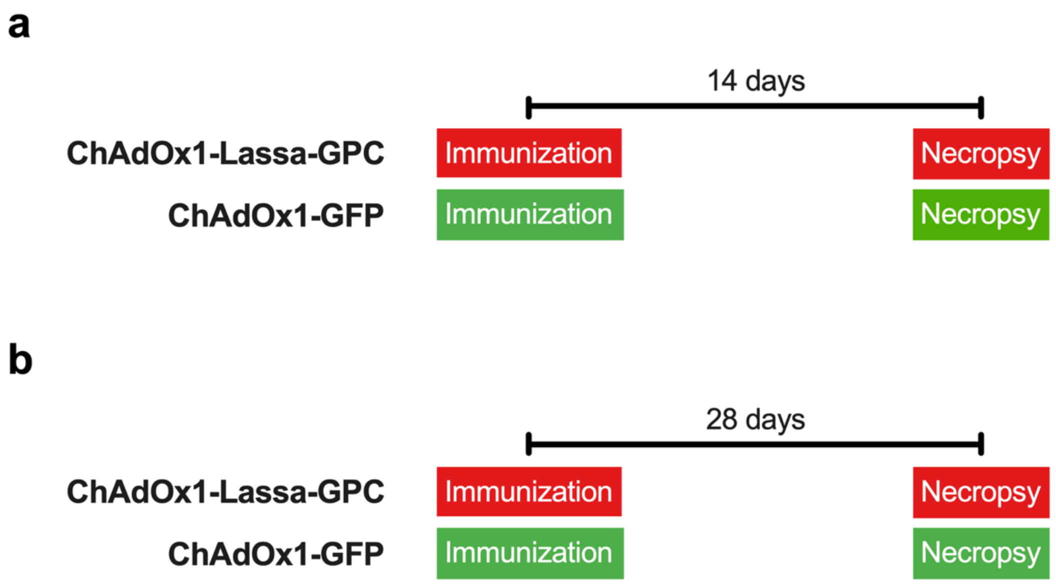

Supplemental Fig. 3. Murine heterosubtypic immunity experimental timelines. (A) For the assessment of T-cell cross-reactivity, mice were immunized with 1.0×10^8 IU of ChAdOx1-Lassa-GPC (n=8) or ChAdOx1-GFP (n=2) in a single dose regimen. All animals were necropsied 14 days after final immunization and spleen samples were collected for analysis. **(B)** For the detection of cross-reactive antibodies, mice were immunized with 1.0×10^8 IU of ChAdOx1-Lassa-GPC (n=8) or ChAdOx1-GFP (n=2) in a single dose regimen. All animals were necropsied 28 days after final immunization and sera was collected for analysis.

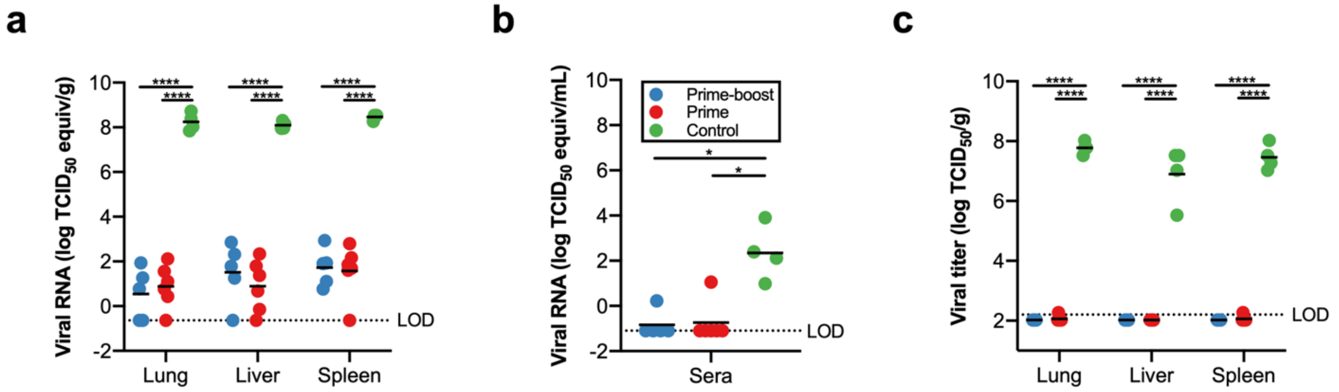

Supplemental Fig. 4. Viral RNA is still present in the tissues of survival cohort animals vaccinated with ChAdOx1-Lassa-GPC at the time of final necropsy but at reduced levels. Lung, liver, spleen, and sera samples were collected from survival cohort animals at final necropsy, which occurred on D28 for vaccinates and (on or before) D12 for controls. Detection of LASV RNA in the tissues **(A)** and sera **(B)** of vaccinated and control animals by qRT-PCR. The dotted lines represent the LOD for tissue (-0.64 log $TCID_{50}$/g equivalents) and sera (-1.10 log $TCID_{50}$/mL equivalents) analysis. Levels of LASV RNA in the lung, liver, and spleen of vaccinated animals were significantly lower than those in controls as determined by two-way ANOVA with Tukey's multiple comparisons test (*****p*<0.0001). Serum viral loads of vaccinated and control animals were significantly different by Kruskal-Wallis non-parametric test with Dunn's multiple comparisons (**p*=0.0192, 0.0169). **(C)** Infectious virus present in the lung, liver, and spleen was quantified via endpoint titration on VeroE6 cells. The dotted line represents the LOD (2.20 log $TCID_{50}$/g). Virus titers in vaccinated animals were significantly reduced compared to control animals as determined by two-way ANOVA with Tukey's multiple comparisons test (*****p*<0.0001).

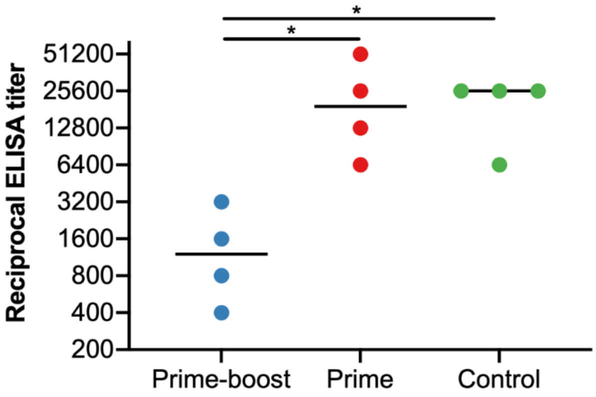

Supplemental Fig. 5. LASV nucleoprotein-specific IgG antibodies are detected in the sera of guinea pigs post challenge regardless of vaccination status. Post-challenge serum samples were collected from animals at the time of necropsy (D12). Anti-LASV nucleoprotein-specific IgG antibody titers in two-fold serial-diluted sera were measured by ELISA. Statistically significant differences in titer (*p=0.0492) were detected between the prime-boost and prime vaccination groups, as well as between the prime-boost and control groups via Kruskal-Wallis nonparametric test and Dunn's multiple comparisons.

Animal Sera	GPA Lassa	VSV-Lassa-GPc-cFLAG
**GP51**	**<10**	**10.67**
**GP52**	**<10**	**10.65**
**GP53**	**<10**	**10.68**
**GP54**	**<10**	**10.66**
**GP55**	**<10**	**10.70**
**GP56**	**<10**	**10.66**
**GP57**	**<10**	**10.63**
**GP59**	**<10**	**10.66**
**GP60**	**<10**	**1.731**

Supplemental Table 1. Sera of prime-boost immunized animals does not exhibit neutralizing activity in live virus and pseudotype-based assays. Sera collected at D0 from animals receiving the prime-boost vaccine regimen was tested for the capacity to neutralize live GPA LASV or VSV-Lassa-GPc-cFLAG. Virus was incubated with sera and added to Vero E6 cells. The cells were cultured with the virus-sera mix for ten days to observe CPE and 16 hours for the detection of GFP-positive infected cells for the live virus and pseudovirus assays, respectively. Neutralizing antibody titers against GPA LASV were estimated using the reciprocal of the maximum serum dilution for which CPE was absent; samples considered to be negative were assigned a titer of <10. Sera neutralizing activity against VSV-Lassa-GPc-cFLAG is represented by IC_{50} values corresponding to the reciprocal sera dilution.
