# Supplementary material for: ChAdOx1-vectored Lassa fever vaccine elicits a robust cellular and humoral immune response and protects guinea pigs against lethal Lassa virus challenge
Source: NPJ Vaccines. 2021 Mar 2;6:32. doi: 10.1038/s41541-021-00291-x (PMC7925663; doi:10.1038/s41541-021-00291-x)
Supplement: Supplementary file 2 — Reporting Summary [file 41541_2021_291_MOESM2_ESM.pdf]

nature research

Corresponding author(s): Munster, NPJVACCINES-00777R

Last updated by author(s): Nov 10, 2020

Reporting Summary

Nature Research wishes to improve the reproducibility of the work that we publish. This form provides structure for consistency and transparency in reporting. For further information on Nature Research policies, see our Editorial Policies and the Editorial Policy Checklist.

Statistics

For all statistical analyses, confirm that the following items are present in the figure legend, table legend, main text, or Methods section.

n/a	Confirmed	
☐	☒	The exact sample size (*n*) for each experimental group/condition, given as a discrete number and unit of measurement
☐	☒	A statement on whether measurements were taken from distinct samples or whether the same sample was measured repeatedly
☐	☒	The statistical test(s) used AND whether they are one- or two-sided<br>*Only common tests should be described solely by name; describe more complex techniques in the Methods section.*
☐	☒	A description of all covariates tested
☐	☒	A description of any assumptions or corrections, such as tests of normality and adjustment for multiple comparisons
☐	☒	A full description of the statistical parameters including central tendency (e.g. means) or other basic estimates (e.g. regression coefficient) AND variation (e.g. standard deviation) or associated estimates of uncertainty (e.g. confidence intervals)
☒	☐	For null hypothesis testing, the test statistic (e.g. *F*, *t*, *r*) with confidence intervals, effect sizes, degrees of freedom and *P* value noted<br>*Give P values as exact values whenever suitable.*
☒	☐	For Bayesian analysis, information on the choice of priors and Markov chain Monte Carlo settings
☒	☐	For hierarchical and complex designs, identification of the appropriate level for tests and full reporting of outcomes
☒	☐	Estimates of effect sizes (e.g. Cohen's *d*, Pearson's *r*), indicating how they were calculated

Our web collection on statistics for biologists contains articles on many of the points above.

Software and code

Policy information about availability of computer code

Data collection	NA
Data analysis	Prism software version 7.04 and 8.0 (GraphPad)

For manuscripts utilizing custom algorithms or software that are central to the research but not yet described in published literature, software must be made available to editors and reviewers. We strongly encourage code deposition in a community repository (e.g. GitHub). See the Nature Research guidelines for submitting code & software for further information.

Data

Policy information about availability of data

All manuscripts must include a data availability statement. This statement should provide the following information, where applicable:

- Accession codes, unique identifiers, or web links for publicly available datasets
- A list of figures that have associated raw data
- A description of any restrictions on data availability

Data will be deposited in Figshare: https://doi.org/10.6084/m9.figshare.12425165.v1

Field-specific reporting

Please select the one below that is the best fit for your research. If you are not sure, read the appropriate sections before making your selection.

☒ Life sciences ☐ Behavioural & social sciences ☐ Ecological, evolutionary & environmental sciences

For a reference copy of the document with all sections, see nature.com/documents/nr-reporting-summary-flat.pdf

Life sciences study design

All studies must disclose on these points even when the disclosure is negative.

Sample size	Sample sizes were determined using power analyses with 95% power
Data exclusions	NA
Replication	Group sizes of 10 animals were used, end point titrations were performed in 4 replicates, neutralization assays were performed in duplicate
Randomization	NA given the specific setting of Biosafety level 4 work
Blinding	NA given the specific setting of Biosafety level 4 work

Reporting for specific materials, systems and methods

We require information from authors about some types of materials, experimental systems and methods used in many studies. Here, indicate whether each material, system or method listed is relevant to your study. If you are not sure if a list item applies to your research, read the appropriate section before selecting a response.

Materials & experimental systems

n/a	Involved in the study	
☐	☒	Antibodies
☐	☒	Eukaryotic cell lines
☒	☐	Palaeontology and archaeology
☐	☒	Animals and other organisms
☒	☐	Human research participants
☒	☐	Clinical data
☒	☐	Dual use research of concern

Methods

n/a	Involved in the study	
☒	☐	ChIP-seq
☐	☒	Flow cytometry
☒	☐	MRI-based neuroimaging

Antibodies

Antibodies used

Anti-mouse CD8a-PerCP/Cy5.5, clone 53-6.7, eBioscience, catalog no. 45-0081-82, dilution 1 in 200
Anti-mouse CD4-BV650, clone RM4-5, BioLegend, catalog no. 100546, dilution 1 in 50
Anti-mouse CD62L-PeCy7, clone MEL-14, eBioscience, catalog no. 25-0621-81, dilution 1 in 50
Anti-mouse CD44-AF700, clone IM7, BioLegend, catalog no. 103026, dilution 1 in 100
Anti-mouse CD127-eF660, clone A7R34, Invitrogen, catalog no. 50-1271-82, dilution 1 in 50
Anti-mouse IFNg-eF450, clone XMG1.2, eBioscience, catalog no. 48-7311-82, dilution 1 in 100
Anti-mouse TNFa-AF488, clone MP6-XT22, eBioscience, catalog no. 53-7321-82, dilution 1 in 100
Anti-mouse IL-2-PE, clone JE56-5H4, eBioscience, catalog no. 12-7021-82, dilution 1 in 100
Alkaline phosphatase-conjugated goat anti-mouse IgG, Sigma, catalog no. A3562, dilution 1 in 5000
KPL peroxidase-labeled antibody to guinea pig (H&L) produced in goat, SeraCare, catalog no. 5220-0366 (14-17-06), dilution 1 in 1000

Validation

All antibodies validated for use in the indicated species by respective suppliers.

Eukaryotic cell lines

Policy information about cell lines

Cell line source(s)	Vero E6 cells
Authentication	Cytochrome B sequencing
Mycoplasma contamination	Tested negative

Commonly misidentified lines (See ICLAC register)

NA

Animals and other organisms

Policy information about studies involving animals; ARRIVE guidelines recommended for reporting animal research

Laboratory animals	Female CD1 mice (Charles River Ltd, Harlow, UK), aged 6-8 weeks, mixed gender Hartley strain guinea pigs (Cavia porcellus), age matched to 14 weeks
Wild animals	NA
Field-collected samples	NA
Ethics oversight	All procedures in CD1 mice were performed as permitted by UK Home Office Project License P9804B4F1 and conducted in accordance with the Animal (Scientific Procedures) Act 1986 with approval by the University of Oxford Animal Care and Ethical Review Committee. LASV challenge experiments in Hartley guinea pigs were approved by the Institutional Animal Care and Use Committee (ACUC) at Rocky Mountain Laboratories (RML).

Note that full information on the approval of the study protocol must also be provided in the manuscript.

Flow Cytometry

Plots

Confirm that:

☒ The axis labels state the marker and fluorochrome used (e.g. CD4-FITC).

☒ The axis scales are clearly visible. Include numbers along axes only for bottom left plot of group (a 'group' is an analysis of identical markers).

☒ All plots are contour plots with outliers or pseudocolor plots.

☒ A numerical value for number of cells or percentage (with statistics) is provided.

Methodology

Sample preparation	A single cell suspension of murine splenocytes was prepared by mechanical disruption of the tissue followed by filtration through a 70 µM cell strainer, erythrocyte lysis with ammonium-chloride-potassium lysing buffer, and resuspension in Minimum Essential Medium Eagle - alpha modification. Cells were stimulated at 37º for 6 hours with a LASV GP peptide pool (2 µg/mL per peptide); BD GolgiPlug was added at a concentration of 1 µg/mL during the last 4 hours of stimulation.
Instrument	BD LSRII
Software	BD FACSDiva Software Version 8.0.2, FlowJo version 10.6.2 for analysis
Cell population abundance	A minimum of 100,000 events were collected within the LIVE/DEAD negative gate. The frequency of CD4+ and CD8+ T cells within the total live cell population ranged from 11.7 - 34.5% and 3.67 - 18.4%, respectively.
Gating strategy	Lymphocytes were isolated based on size using FSC-A vs. SSC-A and doublet discrimination was performed using FSC-A vs. FSC-H. Antigen-specific T cells were identified by gating on LIVE/DEAD negative cells , CD4+ or CD8+ cells, and cytokine positive cells. Memory T cell populations were further distinguished by gating on CD44+ cells and CD62L vs. CD127.

☒ Tick this box to confirm that a figure exemplifying the gating strategy is provided in the Supplementary Information.
